# Dimerization of GPCRs: Novel insight into the role of FLNA and SSAs regulating SST_2_ and SST_5_ homo- and hetero-dimer formation

**DOI:** 10.3389/fendo.2022.892668

**Published:** 2022-08-05

**Authors:** Donatella Treppiedi, Giusy Marra, Genesio Di Muro, Rosa Catalano, Federica Mangili, Emanuela Esposito, Davide Calebiro, Maura Arosio, Erika Peverelli, Giovanna Mantovani

**Affiliations:** ^1^ Department of Clinical Sciences and Community Health, University of Milan, Milan, Italy; ^2^ University Sapienza of Rome, Rome, Italy; ^3^ Institute of Metabolism and Systems Research, University of Birmingham, Birmingham, United Kingdom; ^4^ Centre of Membrane Proteins and Receptors, Universities of Birmingham and Nottingham, Birmingham, United Kingdom; ^5^ Endocrinology Unit, Fondazione Istituto di Ricovero e Cura a Carattere Scientifico (IRCCS) Ca’ Granda Ospedale Maggiore Policlinico, Milan, Italy

**Keywords:** GPCR dimerization, *in situ* PLA, somatostatin analogs, FLNA, SST_2_, SST_5_

## Abstract

The process of GPCR dimerization can have profound effects on GPCR activation, signaling, and intracellular trafficking. Somatostatin receptors (SSTs) are class A GPCRs abundantly expressed in pituitary tumors where they represent the main pharmacological targets of somatostatin analogs (SSAs), thanks to their antisecretory and antiproliferative actions. The cytoskeletal protein filamin A (FLNA) directly interacts with both somatostatin receptor type 2 (SST_2_) and 5 (SST_5_) and regulates their expression and signaling in pituitary tumoral cells. So far, the existence and physiological relevance of SSTs homo- and hetero-dimerization in the pituitary have not been explored. Moreover, whether octreotide or pasireotide may play modulatory effects and whether FLNA may participate to this level of receptor organization have remained elusive. Here, we used a proximity ligation assay (PLA)–based approach for the *in situ* visualization and quantification of SST_2_/SST_5_ dimerization in rat GH3 as well as in human melanoma cells either expressing (A7) or lacking (M2) FLNA. First, we observed the formation of endogenous SST_5_ homo-dimers in GH3, A7, and M2 cells. Using the PLA approach combined with epitope tagging, we detected homo-dimers of human SST_2_ in GH3, A7, and M2 cells transiently co-expressing HA- and SNAP-tagged SST_2_. SST_2_ and SST_5_ can also form endogenous hetero-dimers in these cells. Interestingly, FLNA absence reduced the basal number of hetero-dimers (-36.8 ± 6.3% reduction of PLA events in M2, P < 0.05 *vs*. A7), and octreotide but not pasireotide promoted hetero-dimerization in both A7 and M2 (+20.0 ± 11.8% and +44.1 ± 16.3% increase of PLA events in A7 and M2, respectively, P < 0.05 *vs*. basal). Finally, immunofluorescence data showed that SST_2_ and SST_5_ recruitment at the plasma membrane and internalization are similarly induced by octreotide and pasireotide in GH3 and A7 cells. On the contrary, in M2 cells, octreotide failed to internalize both receptors whereas pasireotide promoted robust receptor internalization at shorter times than in A7 cells. In conclusion, we demonstrated that in GH3 cells SST_2_ and SST_5_ can form both homo- and hetero-dimers and that FLNA plays a role in the formation of SST_2_/SST_5_ hetero-dimers. Moreover, we showed that FLNA regulates SST_2_ and SST_5_ intracellular trafficking induced by octreotide and pasireotide.

## Introduction

G protein-coupled receptors (GPCRs) are the largest family of membrane receptors. Because of their heavy involvement in human physiology and disease, they are major drug targets (1, 2). The biochemical and pharmacological properties of GPCRs have been extensively characterized. One emerging aspect of GPCR signaling is their ability to form homo- and hetero-dimers, which has been implicated in the modulation of ligand binding affinity, signal transduction, and intracellular trafficking (3, 4). Whereas constitutive dimerization is essential for the correct functioning of class C GPCRs (5, 6), its consequences for the function of other GPCRs can vary considerably (7, 8). Of note, hetero-dimerization between two GPCRs of the same or different groups may endow the resulting hetero-dimer with unique signaling and pharmacological properties (9–15).

Somatostatin receptors (SSTs) comprise five family A GPCRs, named SST_1_–SST_5_, which are abundantly expressed in the endocrine system where they exert inhibitory actions on hormone secretion and cell proliferation (16–19). Of these, SST_2_ and SST_5_ are the prevalent subtypes in the pituitary (20, 21). Specifically, SST_2_ and SST_5_ are considered the main pharmacological targets of somatostatin analogs (SSAs) octreotide and pasireotide in the treatment of acromegaly caused by GH-secreting pituitary tumors (22). Octreotide displays a higher binding affinity for SST_2_ (IC_50_ = 0.38 nM), and lower for SST_5_ (6.3 nM), SST3 (7.1 nM), and SST1 (280 nM). On the contrary, pasireotide preferentially binds SST_5_ (0.16 nM) and shows lower affinity for SST_2_ (1 nM), SST_3_ (1.5 nM), and SST_1_ (9.3 nM) (23).

To date, whether SSTs are capable to form homo- and hetero-dimers in the pituitary as well as whether octreotide or pasireotide may play any modulatory effects on these events has not been investigated, yet. Indeed, the field of SST dimerization has been only partially explored in simple cell systems transfected with different SSTs from diverse species (12, 13, 24–27, revised in 28). Moreover, the exact mechanisms governing GPCR dimerization are, with a few exceptions, largely unknown.

An involvement of molecular chaperones such as 14-3-3, HSP70, or receptor activity-modifying proteins (RAMPs) has been proposed (29). Scaffolding proteins and cytoskeletal elements, which are already implicated in the formation of specialized signaling subdomains at the plasma membrane, receptor mobility, cluster assembly, and internalization, may also be involved (30, 31). Among these, the actin-binding protein filamin A (FLNA) might be a good candidate. Thanks to its flexible V shape, its actin-binding domain, and its scaffolding domains, FLNA is involved in crosslinking of actin filaments and anchoring transmembrane receptors to the subcortical cytoskeleton, thus providing a scaffold platform for receptor spatial organization and signaling (32). By means of single-molecule imaging and *in situ* proximity ligation assay (PLA), we have previously demonstrated that SST_2_ and SST_5_ directly interact with FLNA (33, 34). In pituitary tumoral cell lines, the association of FLNA with both SST_2_ and SST_5_ is crucial for an efficient SSA-induced signal transduction (34, 35). In addition, FLNA expression is required for SST_2_ internalization, recycling to the plasma membrane and protein stability after prolonged agonist stimulation (35), and to maintain a stable amount of SST_5_ in basal conditions by preventing both lysosomal and proteosomal degradation (34). However, whether FLNA may participate to SST dimeric assembly remains to be assessed.

In the present work, we used a PLA-based methodology for the *in situ* visualization and quantification of SST_2_/SST_5_ dimerization in rat GH-secreting pituitary cells (GH3). Moreover, in order to investigate the role of FLNA, we used the human melanoma cell models either expressing (A7) or lacking (M2) FLNA.

*In situ* PLA represents a powerful strategy to study receptor dimerization also in its natural context at physiological expression levels (36, 37). Moreover, we performed immunofluorescence experiments to demonstrate the specific FLNA-dependent modulation of SST_2_ and SST_5_ intracellular trafficking induced by octreotide and pasireotide.

## Materials and methods

### Cell culture

Rat pituitary tumoral GH-secreting cells (GH3 cells, ATCC CCL-82.1) were purchased from ATCC (Manassas, VA, USA) and cultured in F12K medium supplemented with 15% horse serum (HS), 2.5% fetal bovine serum (FBS), and antibiotics (Life Technologies, Carlsbad, CA, USA).

M2 is a spontaneously FLNA-deficient cell line, established from the tumor of a patient with malignant melanoma (38). A7 is a stably transfected cell line derived from the M2 cell line. A7 and M2 cells were kindly provided by Dr. Fumihiko Nakamura (School of Pharmaceutical Science and Technology, Tianjin University, Tianjin, China) and were grown in Eagle’s Minimum Essential Medium (EMEM) (ATCC, Manassas, VA, USA) supplemented with 8% Newborn Calf serum (NBCS), 2% fetal bovine serum (FBS), and antibiotics (Life Technologies, Carlsbad, CA, USA). For A7 cells, 200 µg/ml G418 was added (Merck KGaA, Darmstadt, DE). Cells were kept at 37°C in a humidified atmosphere with 5% CO_2_. Octreotide and pasireotide were provided by Novartis Pharma AG (Basel, CH) and used at 100-nM concentration.

### Plasmid transfection

Expression vectors coding for human HA-SSTR2 (influenza hemagglutinin-tagged SSTR2) and SNAP-SSTR2 (SSTR2 fused to SNAP tag, a 20-kDa protein derived from the enzyme *O* ^6^-alkylguanine-DNA alkyltransferase) were previously described (33). These vectors were transiently co-transfected in GH3 cells for 6 h in order to achieve low expression levels, resembling those of endogenous SSTR2. Lipofectamine 2000 was used as transfection reagent (Invitrogen, Thermo Fisher Scientific, Waltham, MA, USA) according to the manufacturer’s instruction.

### *In situ* proximity ligation assay

GH3, A7, and M2 cells were seeded on 13-mm poly-L-lysine-coated coverslips at a density of 1.25 × 10^5^ cells/well in 24-well plates and grown at 37°C for 18 h. The following day, cells were exposed or not with pasireotide 100 nM or octreotide 100 nM for 5 min. In case of SSTR2/SSTR2 homo-dimer evaluation, cells were transiently transfected with HA-tagged SSTR2 and SNAP-tagged SSTR2, as described above. Cells were fixed with 4% paraformaldehyde (Merck KGaA, Darmstadt, DE) for 10 min at room temperature, washed three times with PBS, and incubated for 1 h at room temperature with blocking buffer (5% FBS, 0.3% Triton™ X-100, in PBS). To test the presence of SST_2_/SST_5_ hetero-dimers, coverslips were incubated overnight at 4°C with primary rabbit anti-SST_2_ UMB1 #ab134152 (1:50, Abcam, Cambridge, UK) and primary mouse anti-SST_5_ #6675-1-Ig (1:200, Proteintech, Rosemont, IL, USA) antibodies. To test the presence of SST_5_/SST_5_ homo-dimers, two different primary antibodies against SST_5_ were used: a rabbit anti-SST_5_ #PA3-209 (Thermo Fisher Scientific, CA, USA) and a mouse anti-SST_5_ #6675-1-Ig (Proteintech, Rosemont, IL, USA), both diluted 1:200. To test the presence of SST_2_/SST_2_ homo-dimers, primary mouse anti-HA #26183 (1:250, Thermo Fisher Scientific, CA, USA) and primary rabbit anti-SNAP #CAB4255 (1:800, Thermo Fisher Scientific, CA, USA) antibodies were used. All antibodies were diluted in antibody Diluent Reagent Solution (Life Technologies, Thermo Fisher, CA). As negative controls to detect potential unspecific signal, one of the primary antibodies was removed. We used the Duolink *In Situ* PLA kit from Sigma-Aldrich (Merck KGaA, Darmstadt, DE). Briefly, Duolink Anti-Rabbit PLUS Probe (DUO92002, Sigma-Aldrich) and Duolink Anti-Mouse MINUS Probe (DUO92040, Sigma-Aldrich) were added and incubated for 1 h at 37°C. Then, Duolink *In Situ* Detection Reagents Green (Duolink, DUO92014) was used. Ligation-Ligase solution was added and incubated for 30 min at 37°C. Amplification-polymerase solution was subsequently added and incubated for 2 h (for SST_2_/SST_2_ homo-dimer detection) or 18 h (for SST_2_/SST_5_ hetero-dimer and SST_5_/SST_5_ homo-dimer detection) at 37°C. Coverslips were mounted on glass slides with EverBrite™ Hardset Mounting Medium with DAPI (Biotium, Fremont, CA, USA) for subsequent observation under epifluorescence microscope. Proximity ligation events were quantified with NIH ImageJ software after image deconvolution. The average number of PLA puncta per cell are related to images acquired from randomly chosen fields per condition from three independent experiments and quantified as previously described (34).

### Immunofluorescence

GH3, A7, and M2 cells were seeded on 13-mm poly-L-lysine-coated coverslips at a density of 1.25 × 10^5^ cells/well in 24-well plates and grown at 37°C for 18 h. The following day, cells were incubated with pasireotide 100 nM or octreotide 100 nM for 0, 5, or 30 min. For immunofluorescence analysis of SST_2_ and SST_5_ localization in GH3, A7, and M2 cells, rabbit anti-SST_2_ UMB1 #ab134152 (1:50, Abcam, Cambridge, UK) and mouse anti-SST_5_ #6675-1-Ig (1:200, Proteintech, Rosemont, IL, USA) antibodies were used and incubated o/n at 4°C. Anti-mouse Alexa Fluor™ 546-conjugated secondary antibody (1:500, Thermo Fisher Scientific, CA, USA) and anti-rabbit Alexa Fluor™ 488-conjugated secondary antibody (1:500, Thermo Fisher Scientific, CA, USA) were incubated at room temperature for 2 h. All antibodies were diluted in Antibody Diluent Reagent Solution (Life Technologies, Thermo Fisher, CA, USA). Coverslips were mounted on glass slides with EverBrite™ Hardset Mounting Medium with DAPI (Biotium, Fremont, CA, USA) for subsequent observation under epifluorescence microscope. The NIH ImageJ software was used to merge single-channel images before and after deconvolution.

### Statistical analysis

The results are expressed as the mean ± SD. A paired two-tailed Student’s t-test was applied to assess the significance between two series of data. Statistical analysis was performed by GraphPad Prism 7.0 software, and *P* < 0.05 was accepted as statistically significant.

## Results

### Detection of SST_2_/SST_2_ and SST_5_/SST_5_ homodimers in somatotroph and melanoma cells

In order to examine the occurrence of SST_2_/SST_2_ homo-dimers in rat pituitary GH-secreting cells (GH3) and human melanoma cells A7 (FLNA-expressing cells) and M2 (FLNA-lacking cells), we combined the PLA approach with epitope tagging. Cells were transiently co-transfected with human HA-tagged SST_2_ and SNAP-tagged SST_2_, and antibodies against HA and SNAP tags were then used for PLA experiments. Indeed, in preliminary experiments, we did not find a pair of anti-SST_2_ antibodies raised in two different species, suitable for this application. To detect endogenous SST_5_/SST_5_ homo-dimers in GH3, A7, and M2 cells, two separate anti-SST_5_ antibodies raised in mouse and rabbit were used.

**Figure 1:**
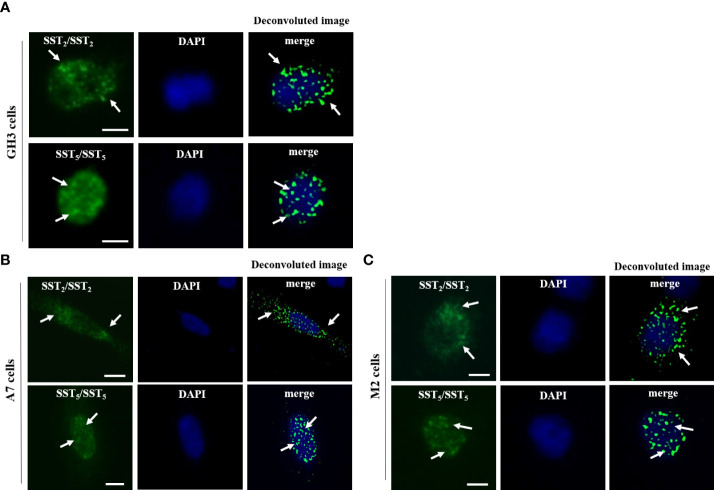
In situ detection of SST2/SST2 and SST5/SST5 homo-dimers. Representative *in situ* PLA experiment performed in GH3 **(A)**, A7 cells **(B)**, and M2 cells **(C)** showing SST_2_/SST_2_ (upper panels) and SST_5_/SST_5_ (lower panels) homo-dimers. For SST_2_/SST_2_ homo-dimers, mouse anti-HA and rabbit anti-SNAP antibodies were used. For SST_5_/SST_5_ homo-dimers, mouse and rabbit anti-SST_5_ antibodies were used. PLA puncta representing homo-dimers are shown as green dots and nuclei are stained with DAPI in blue. A deconvoluted image of PLA events merged with DAPI is shown. White arrows indicated the localization of PLA puncta. Scale bars: 10 μm.

In GH3 cells, SST_2_/SST_2_ homo-dimers and SST_5_/SST_5_ homo-dimers were detected under basal conditions (**Figure 1A**). The presence of SST_2_/SST_2_ homo-dimers and SST_5_/SST_5_ homo-dimers were observed in both A7 and M2 cells, regardless of FLNA expression (**Figures 1B, C**). There were no SST_2_/SST_2_ or SST_5_/SST_5_ dimer signals in negative controls (**Supplementary Figure 1**).

### Effect of SSAs and FLNA on SST_2_/SST_5_ hetero-dimerization in somatotroph and melanoma cells

**Figure 2:**
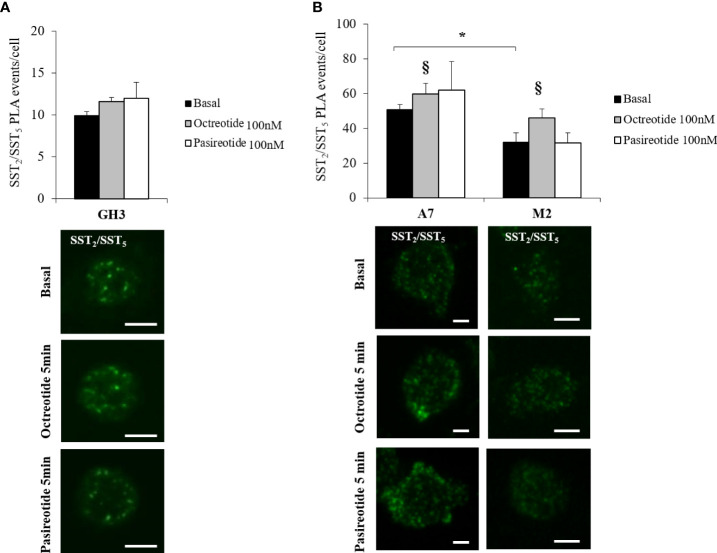
In situ detection of SST2/SST5 hetero-dimers. Representative *in situ* PLA experiment showing SST_2_/SST_5_ hetero-dimers in GH3 **(A)** and melanoma cells **(B)** before and after treatments with 100 nM octreotide or pasireotide for 5 min. Rabbit anti-SST_2_ and mouse-anti SST_5_ antibodies were used. Green dots represent PLA events and indicate close proximity between SST_2_ and SST_5_. Graphs resulting from the quantification of total SST_2_/SST_5_ puncta representing PLA events are shown for each cell line. For **(B)**, a reduction in basal SST_2_/SST_5_ hetero-dimers in M2 cells compared to A7 cells and an increase in SST_2_/SST_5_ hetero-dimers in A7 and M2 cells treated with octreotide compared to basal are shown (n = 3, number of PLA puncta per cells was quantified for 150 cells randomly chosen from different fields per condition, *p < 0.05 *vs*. basal A7 cells; ^§^p < 0.05 *vs*. corresponding basal). Scale bars: 10 μm.

Then, we investigated the presence of endogenous SST_2_/SST_5_ hetero-dimers in GH3 cells by means of PLA analysis and tested the possible effects exerted by octreotide and pasireotide on this receptor dimeric state. As shown in **Figure 2A**, SST_2_/SST_5_ hetero-dimers were detected under basal conditions and after treatment with 100 nM octreotide or pasireotide for 5 min. No effect on the number of PLA puncta corresponding to SST_2_/SST_5_ hetero-dimers was observed.

To decipher the role of FLNA in the formation of SST_2_/SST_5_ hetero-dimers and in the modulation of SSA-dependent effects on receptor assembly, PLA experiments were repeated in A7 and M2 cells. Our results showed that SST_2_ and SST_5_ were able to form hetero-dimers in A7 and M2 cells under basal physiological conditions. However, the absence of FLNA significantly impaired the amount of SST_2_/SST_5_ hetero-dimers (-36.8 ± 6.3% reduction of PLA events in M2 cells, P < 0.05 *vs*. A7 cells). In both cell lines, a significant increase in the PLA events was observed after 5 min of incubation with octreotide (+20.0 ± 11.8% increase of PLA events in A7 cells, P < 0.05 *vs*. basal, and +44.1 ± 16.3% increase of PLA events in M2 cells, P < 0.05 *vs*. basal) but not pasireotide (**Figure 2B**). No SST_2_/SST_5_ dimer signals were detected in negative controls (**Supplementary Figure 2**).

### FLNA-dependent modulation of SSA-induced intracellular trafficking of SST_2_ and SST_5_

**Figure 3:**
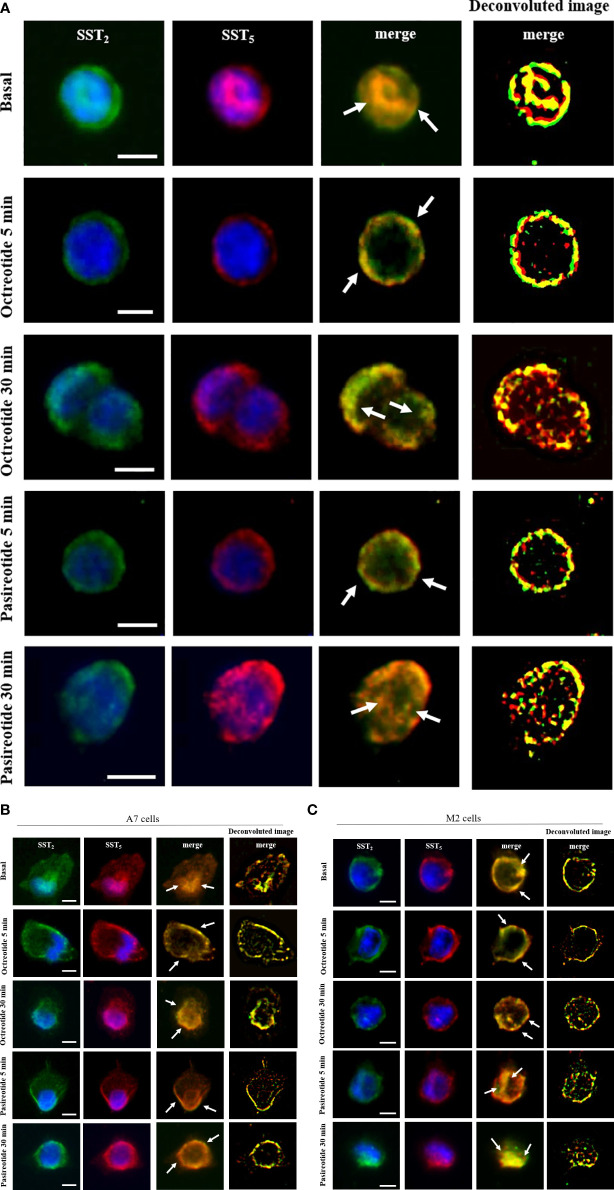
SST2 and SST5 intracellular trafficking. Representative immunofluorescence experiment showing subcellular localization of SST_2_ (green) and SST_5_ (red) in GH3 cells **(A)**, A7 cells **(B)**, and M2 cells **(C)** stimulated or not with 100 nM octreotide or pasireotide for the indicated times. Nuclei are stained with DAPI in blue. Overlay of green and red channels is shown, and white arrows indicate SST_2_ and SST_5_ subcellular colocalization. Deconvolution algorithm was applied to further show SST_2_ and SST_5_ colocalization areas (yellow signals, right columns). Scale bars: 10 μm.

Next, we used immunofluorescence to follow SSA-induced intracellular trafficking of endogenous SST_2_ and SST_5_ in GH3. Our imaging data showed that SST_2_ and SST_5_ colocalize throughout the cell body in the absence of stimuli. Cells’ exposure to octreotide or pasireotide for 5 min resulted in rapid receptor translocation to the plasma membrane. SST_2_ and SST_5_ were then similarly internalized by longer-time stimulation with octreotide and pasireotide, as shown by the intracellular colocalization signal (**Figure 3A**).

To investigate the role of FLNA in the colocalization of human SST_2_ and SST_5_ during internalization experiments, A7 and M2 cells were used. Under basal conditions, SST_2_ and SST_5_ share the same localization at intracellular sites and on the plasma membrane in both cell lines. In A7 cells, stimulation with 100 nM octreotide and pasireotide induced a rapid cell membrane recruitment of both receptors (at 5 min) and receptor accumulation in a perinuclear region (at 30 min) (**Figure 3B**). In M2 cells, whereas a 5-min exposure to octreotide resulted in complete SST_2_ and SST_5_ translocation to the plasma membrane, similarly to what is observed in A7 cells, pasireotide also promoted a rapid internalization of a subset of both receptors. After 30 min of stimulation, only pasireotide led to SST_2_ and SST_5_ accumulation in a perinuclear region, while octreotide failed to internalize both receptors, as shown by the persistence of membrane signals (**Figure 3C**).

## Discussion

SST_2_ and SST_5_ are the most abundantly expressed SSTs in the pituitary, where they mediate octreotide and pasireotide inhibitory effects on hormone secretion and cell proliferation, representing the main pharmacological targets for GH-secreting pituitary tumors in acromegaly (22). FLNA is known to directly bind SST_2_ and SST_5_ influencing their expression, signaling, and intracellular trafficking (34–36). To date, the co-expression of SST_2_ and SST_5_ has been reported in pituitary tumor cells (17, 39). However, the occurrence of SSTs homo- and hetero-dimerization and the modulation by SSAs as well as a possible involvement of FLNA in this process have been only postulated (40, 41). In the present study, we provided evidence of SST_2_ and SST_5_ homo and hetero-dimer assembly in pituitary somatotroph GH3 cells and melanoma cells and the impact of FLNA and octreotide, but not pasireotide, in the modulation of SST_2_/SST_5_ hetero-dimer formation. Moreover, we uncovered a novel role of FLNA in SSA-dependent SST_2_ and SST_5_ internalization.

First, by *in situ* PLA we detected the presence of homo-dimers of transiently transfected human SST_2_ in GH3 cells. This finding is consistent with previous immunoprecipitation and fluorescence resonance energy transfer (FRET) results on human, rat, and porcine SST_2_ in transfected in CHO and HEK293 cells, which revealed interspecies variations in the response to somatostatin (13, 24, 26). Here, we showed that SST_2_/SST_2_ homo-dimer assembly also occurs in melanoma cells. Moreover, our results suggest an involvement of FLNA-independent processes, since both A7 and M2 cell lines displayed PLA signals under basal conditions. As regards SST_5_, our PLA experiments showed that endogenous rat and human receptors assemble to form homo-dimers in GH3 and melanoma cells both under basal and stimulated conditions, respectively. This is in apparent contrast to previous studies which suggested that human SST_5_ does not dimerize after its synthesis but rather following somatostatin treatment (25). However, there is evidence that the stringent protein solubilization conditions used to study dimerization with co-immunoprecipitation may induce dimer dissociation, whereas agonist binding may stabilize receptor dimers (42). Since PLA does not require protein solubilization and has a higher sensitivity, it is plausible that our approach was able to reveal constitutive SST_5_ dimers that could not be detected in previous biochemical studies.

Since SST_5_/SST_5_ homo-dimers were present in both A7 and M2 cells, our results rule out a role of FLNA in SST_5_ homo-dimerization. Unfortunately, we could not perform a quantitative analysis of possible effects exerted by SSAs on SST_2_/SST_2_ and SST_5_/SST_5_ homo-dimers due to technical limitations. Specifically, in the case of SST_2_/SST_2_ homo-dimers, the difference in SST_2_ transfection efficiency among cells represented a bias, that, in turn, could have affected the PLA outcome; in the case of SST_5_/SST_5_ homo-dimers, the PLA coalescent signal (a consequence of the use of two different antibodies directed toward the same protein) could have rendered the puncta counting step unreliable.

Regarding GPCR hetero-dimerization, this process may be critical for the correct functionality of the GPCR, as is the case of the and the γ-aminobutyric acid (GABA_B_) receptor (5, 6), or, in some instances, may result in the alteration of the single receptor functioning (9–15). Here, the occurrence of endogenous SST_2_/SST_5_ hetero-dimers was reported for GH3 cells, although they remained insensitive to octreotide and pasireotide stimulation. In melanoma cells, SST_2_/SST_5_ hetero-dimers were also observed, but, interestingly, the absence of FLNA resulted in a significant reduction of their amount under basal conditions, indicating that FLNA is required but not essential for bringing SST_2_ and SST_5_ in close proximity to dimerize. Hetero-dimer formation was enhanced by octreotide but not pasireotide in both A7 and M2 cells, suggesting that activation of SST_2_ but not SST_5_ is an FLNA-independent driving factor for receptors to assemble. It has to be noticed that the effect of octreotide in M2 cells seemed even more pronounced. This observation raises the hypothesis of a less organized and more random occurrence of receptor interactions in the absence of FLNA. Indeed, in previous single-molecule studies, the disrupted FLNA–SST_2_ interaction resulted in upregulation of freely diffusing SST_2_ in CHO cells (33). However, in line with our finding of octreotide-promoting SST_2_/SST_5_ hetero-dimerization, co-immunoprecipitation and FRET data published by Grant and colleagues already documented an upregulation of human SST_2_/SST_5_ hetero-dimers in HEK293 cells following the selective activation of SST_2_ but not SST_5_ or their co-stimulation, with implications on receptor dynamics such as association of β-arrestin to SST_2_, receptor recycling, and signaling transduction efficiency (27).

An issue that should be considered is that the experiments testing SST_2_/SST_2_ homo-dimerization were performed by transient transfection of human SST2 in rat GH3 cells, since a pair of anti-SST_2_ antibodies raised in two different species, suitable for this application, and a human tumoral pituitary cell line were unavailable. Admittedly, a limitation of the present study is the lack of primary cell cultures from GH-secreting pituitary tumors.

Moreover, further studies analyzing a possible differential function of dimers compared to receptor monomers in terms of enhanced response to SSA treatment or activation of different intracellular pathways will open the way for the development of new therapeutic strategies for pituitary tumors based on drugs targeting the formation of SST dimers.

Finally, we studied SST_2_ and SST_5_ trafficking in order to highlight a potential role of FLNA in the modulation of ligand-mediated receptor internalization. We first observed a similar efficiency of octreotide and pasireotide in the recruitment of SST_2_ and SST_5_ at the plasma membrane and subsequent internalization in GH3 cells and A7 cells, supporting the idea of an efficient activation of these receptors exerted by both compounds in these specific cell models. These findings were only partially in line with data present in the literature reporting different effects on SST dynamics promoted by octreotide and pasireotide (43, 44). As already reported for SST_2_ in experiments of FLNA silencing in somatotroph cells (45), here we observed a lesser extent of octreotide-induced internalization not only of SST_2_ but also of SST_5_ in M2 cells compared to A7 cells. In addition, we documented a faster mobilization and internalization of both SST_2_ and SST_5_ in M2 cells compared to A7 cells promoted by pasireotide. These data pointed out a specific FLNA-dependent modulation of SST_2_ and SST_5_ intracellular trafficking induced by octreotide and pasireotide.

In conclusion, this work provides novel insights into the molecular mechanisms modulating SST assembly and functioning. Although the SST dimer interfaces remain to be established, such studies may open the way for the design of drugs targeting specific interactions between receptors of the SSTs family as well as interaction between scaffold proteins and SSTs.

## Data availability statement

The original contributions presented in the study are included in the article/**Supplementary Material**. Further inquiries can be directed to the corresponding author.

## Author contributions

Conceptualization: EP and GMan; investigation: DT, GMar, GeM, RC, FM, and EE; software: DT; writing—original draft preparation: DT; writing—review and editing: EP, DC, and GMan; supervision: MA; funding acquisition: EP, DC, and GMan. All authors contributed to the article and approved the submitted version.

## Funding

This work was supported by the Italian Ministry of Health grant to GM (PE-2016-02361797), by an AIRC (Associazione Italiana Ricerca Cancro) grant to GM (IG 2017–20594), and by a Progetti di Ricerca di Interesse Nazionale (PRIN) grant to EP (2017N8CK4K). DC is a recipient of a Wellcome Trust Senior Research Fellowship (212313/Z/18/Z).

## Conflict of interest

The authors declare that the research was conducted in the absence of any commercial or financial relationships that could be construed as a potential conflict of interest.

## Publisher’s note

All claims expressed in this article are solely those of the authors and do not necessarily represent those of their affiliated organizations, or those of the publisher, the editors and the reviewers. Any product that may be evaluated in this article, or claim that may be made by its manufacturer, is not guaranteed or endorsed by the publisher.
