# Supplementary figures and images for: Dimerization of GPCRs: Novel insight into the role of FLNA and SSAs regulating SST2 and SST5 homo- and hetero-dimer formation
Source: Front Endocrinol (Lausanne). 2022 Aug 5;13:892668. doi: 10.3389/fendo.2022.892668 (PMC9389162; doi:10.3389/fendo.2022.892668)

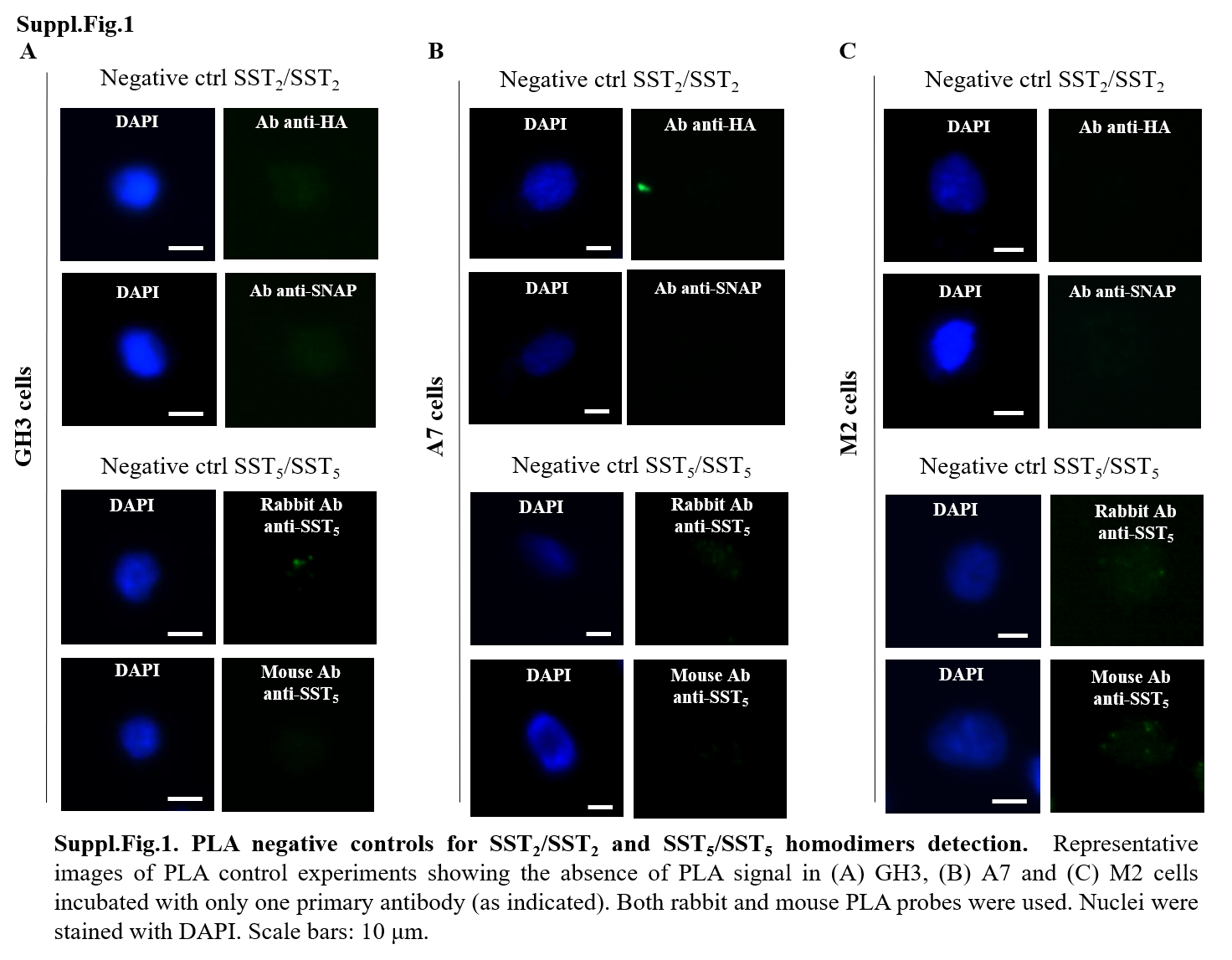

Supplement: Supplementary file 1 [file Image_1.tif]

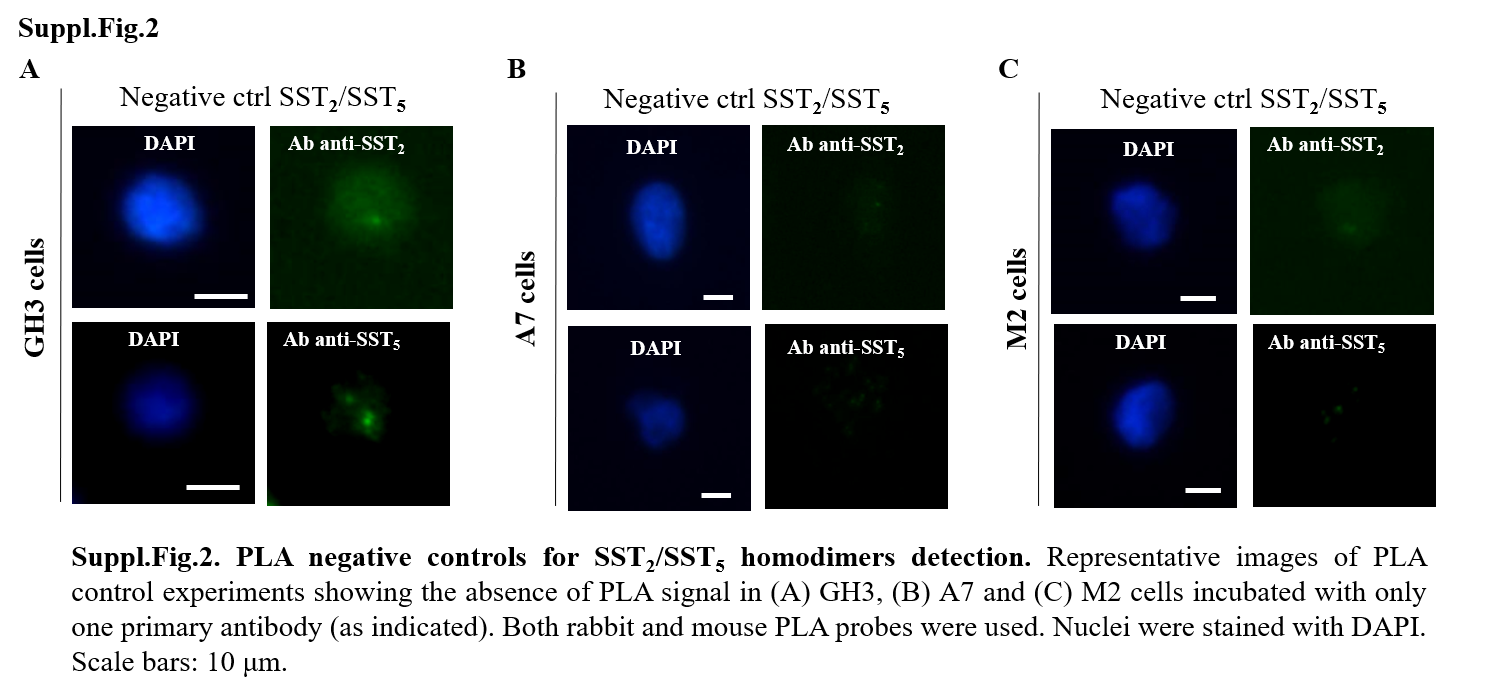

Supplement: Supplementary file 2 [file Image_2.tif]
